# Rare and severe complications of congenital adrenal hyperplasia due to 21-hydroxylase deficiency: a case report

**DOI:** 10.1186/1752-1947-7-39

**Published:** 2013-02-06

**Authors:** Florbela Ferreira, João Martin Martins, Sónia do Vale, Rui Esteves, Garção Nunes, Isabel do Carmo

**Affiliations:** 1 Endocrine and Metabolic Department, Santa Maria Hospital and Lisbon Medical School, Lisbon, Portugal; 2 Department of Surgery, Santa Maria Hospital, Lisbon, Portugal; 3 Department of Urology, Curry Cabral Hospital, Lisbon, Portugal

**Keywords:** 21-hydroxylase, Adrenal myelolipoma, Adrenogenital rests, Congenital adrenal hyperplasia

## Abstract

**Introduction:**

We report the case of a patient with classical congenital adrenal hyperplasia due to 21-hydroxylase deficiency who presented with unusual anatomical and biochemical features, namely massively enlarged adrenal glands, adrenogenital rest tissue and an unexpected endocrine profile. The contribution of the adrenocortical cells in the adrenals and testicles was determined by a cosyntropin stimulation test before and after adrenalectomy. To the best of our knowledge this is the first report of such a case in the literature.

**Case presentation:**

A 35-year-old Caucasian man was admitted to the emergency room with an Addisonian crisis. He had been diagnosed with congenital adrenal hyperplasia in the neonatal period. He acknowledged poor adherence to treatment and irregular medical assistance. Physical examination revealed marked cutaneous and gingival hyperpigmentation, hypotension, and hard nodules in the upper pole of both testicles. Blood analysis showed mild anemia and hyponatremia and no evidence of acute infection. Endocrine evaluation showed very low cortisol levels, low dehydroepiandrosterone-sulfate and elevated corticotropin, 11-deoxycortisol and delta-4-androstenedione. The concentration of 17-hydroxyprogesterone was 20,400ng/dL. After the cosyntropin stimulation test the pattern was similar and there was no significant increase in cortisol or 17-hydroxyprogesterone. The abdominal computed tomography scan revealed grossly enlarged and heterogeneous adrenal glands (left, 12cm; and right, six cm). A bilateral adrenalectomy was performed and pathologic examination revealed adrenal myelolipomas with nodular cortical hyperplasia. The sonogram showed bilateral heterogeneous masses on the upper pole of both testes which corresponded to the nodular hyperplasia of adrenal rest tissues. The genetic study revealed compound heterozigoty (mutations R124H and R356W), suggestive of a phenotypically moderate disease. We performed a cosyntropin stimulation test after adrenalectomy. The steroidogenic profile displayed the same unusual features, indicating an important contribution from the adrenogenital cells.

**Conclusion:**

This case illustrates that congenital adrenal hyperplasia due to 21-hydroxylase deficiency can progress to severe acute and chronic complications. The masses in the patient’s adrenal glands and testicles resulted from chronically elevated adrenocorticotropic hormone and growth of adrenocortical cells. The basal and stimulated steroid profile, before and after adrenalectomy, revealed an unexpected pattern, suggesting significant contribution of the testicular adrenal cells to the steroidogenesis.

## Introduction

Congenital adrenal hyperplasia (CAH) is a group of inherited autosomal recessive disorders characterized by a defect in one of the five enzymes responsible for cortisol biosynthesis. The most common type of CAH, responsible for 90% to 95% of all cases is 21-hydroxylase (21-OH) deficiency [1,2].

The enzyme 21-OH converts progesterone to deoxycorticosterone and 17-hydroxyprogesterone (17-OHP) to 11-deoxycortisol. When the enzyme is absent or deficient, the precursors in these enzymatic reactions accumulate; this is particularly true of 17-OHP [2,3] and, to a lesser degree, progesterone, 17-hydroxypregnenolone, and androstenedione [4]. The latter, which is converted peripherally to testosterone, is responsible for the androgenic signs and symptoms characteristic of CAH. The incidence of classical 21-OH deficiency varies between 1:11800 and 1:21800, according to the population background [2]; the carrier frequency is 1:50 to 1:60 in Western countries [1,2].

There is a wide spectrum of phenotypes. The classical disease manifests early in life and is subdivided into a salt-wasting form, which is more severe, and a simple virilizing form. The milder, non-classical, disease manifests later in life, with various degrees of virilization and infertility in female patients [1,2,4]. About 75% of patients who have classical 21-OH deficiency cannot synthesize adequate amounts of aldosterone [3,4]. The combined deficiency of cortisol and aldosterone causes hyponatremic dehydration and shock in inadequately treated patients; this is termed “salt-wasting crisis” [3].

Because of overproduction of androgens during fetal life, female patients with classical 21-OH deficiency exhibit virilization of the external genitalia at birth [1,2], late menarche, infertility and masculine behavioral and cognitive traits [4]. Fertility can also be affected in male patients who are 21-OH deficient because of inadequate spermatogenesis and development of testicular adrenal rest tissues [3,5]. In both genders, linear growth is affected, with consequent short stature [3].

The aim of early diagnosis and treatment in 21-OH deficiency is to provide cortisol equivalent to that which is normally produced and to suppress adrenal androgens without impairing growth [5,6] and normal pubertal development and fertility. Prenatal diagnosis and *in utero* dexamethasone treatment in female patients and lifelong hydrocortisone and fludrocortisone replacement after birth can prevent all of the manifestations. If therapy is withheld, low cortisol levels and persistently elevated adrenocorticotropic hormone (ACTH) can lead to serious acute and chronic complications.

## Case presentation

A 35-year-old Caucasian man, with the previous diagnosis of CAH, was admitted to the Emergency department of a central hospital in Lisbon, Portugal, with anorexia, nausea, vomiting and abdominal pain. He reported feeling ill for four weeks, since he had an upper respiratory tract infection. In the previous three days he was feeling extremely tired, had persistently vomited and had epigastric pain. At admission he was dehydrated and hypotensive (blood pressure 95/48mmHg). He weighed 73kg (height 173cm; body mass index 24.4kg/m^2^). Abdominal palpation elicited moderate pain in the epigastrium, but no masses were detected. There was cutaneous hyperpigmentation, not only in sun-exposed areas but also in the axillary and genital areas and gingiva. His genitals were stage 5 of the Tanner scale. He had no facial or body acne. The distribution of body hair and muscular development was normal for his age and sex. On palpation, both testicles presented a petrous, irregularly defined mass (approximately 3x3cm) on the upper pole.

Blood tests showed mild anemia (hemoglobin 11.0g/dL) and sodium (132mmol/L) and serum osmolality (270mOsm/kg) were slightly below the normal range. Serum glucose and potassium levels were normal and there was no evidence of infection. The electrocardiogram record and chest roentgenogram were normal.

CAH had been diagnosed in the neonatal period, in Dusseldorf, Germany, after a seizure and transient coma. Daily treatment with glucocorticoids (prednisone) and mineralocorticoids (fludrocortisone) was started (no record of the dosage could be obtained). The patient was instructed to follow a hypersaline diet. Despite alleged compliance to therapy, he had multiple hospital admissions during childhood. Puberty occurred at the age of 12, with normal development of the external genitalia and secondary sex characteristics and his final height at adulthood was within the normal range for gender and genetic potential, as calculated from his parents’ adult height (mother 161cm, father 178cm; target height 176±8cm). During adulthood he was prescribed hydrocortisone at 20mg a day in a single dosage. He was instructed to double the hydrocortisone daily dosage in situations of acute stress. For the past 10 years there was no regular outpatient follow-up and compliance with therapy was irregular, but there were no hospital admissions due to acute adrenal insufficiency until the reported episode.

His past medical history was irrelevant except for the diagnosis of epilepsy at the age of three. He was treated since then with sodium valproate and carbamazepine. No CAH or other endocrine abnormalities had been diagnosed in his siblings or other family relatives. There was no record of consanguinity in his first degree relatives.

Venous blood was drawn for a complete endocrine panel before starting corticoid replacement therapy (Table 1). Measurements of carcinogenic embryonic antigen (CEA), alpha-fetoprotein (α-FP), and beta-human chorionic gonadotropin (β-HCG) were negative (data not shown). The cosyntropin test was performed on the third day of hospital admission, more than eight hours after the last administration of intravenous hydrocortisone. Blood was drawn at baseline, and at 30 and 60 minutes after intravenous injection of 250μg cosyntropin (Table 2).

**Table 1:** Serum and urinary hormonal levels – basal conditions

	**Result**	**RV ♂**		**Result**	**RV ♂**
**ACTH**	480	0–46pg/mL	**FSH**	<0.30	1.4–18.1U/L
**Cortisol**	3	4.3–23μg/dL	**LH**	<0.07	1.5–9.3U/L
**17-OHP**	204.0	0.6–3.4ng/mL	**Estradiol**	<10.0	<50pg/mL
**DHEAS**	39.4	80–560μg/dL	**11-deoxycortisol**	5.6	<7.2ng/mL
**Delta-4-androstenedione**	>10.0	0.6–3.1ng/mL	**Renin**	37.8	1–20pg/mL
**Total testosterone**	782.0	241–827ng/dL	**Aldosterone**	147.1	10–160pg/mL
**Free testosterone**	10.8	8.8–27pg/mL			

ACTH, Adrenocorticotropic hormone; DHEAS, Dehydroepiandrosterone-sulfate; FSH, Follicle-stimulating hormone; LH, Luteinizing hormone; 17-OHP, 17-hydroxyprogesterone; RV ♂, Reference values for the male population.

**Table 2:** Results of the cosyntropin stimulation test (basal, 30 and 60 minutes post-cosyntropin)

	**RV ♂**	**0 min**	**30 min**	**60 min**
**ACTH**	0–46pg/mL	319		
**Cortisol**	4.3–23μg/dL	2	3	3
**17-OHP**	0.6–3.4ng/mL	222	214	177
**11-deoxycortisol**	<7.2ng/mL	20	44.4	55.6
**Delta-4-androstenedione**	0.6–3.1ng/mL	19	30	29
**DHEAS**	80–560μg/dL	26	36	47
**Total testosterone**	241–827ng/dL	702	1070	1032

ACTH, Adrenocorticotropic hormone; DHEAS, Dehydroepiandrosterone-sulfate; 17-OHP, 17-hydroxyprogesterone; min, Minute; RV ♂, Reference values for the male population.

**Figure 1:**
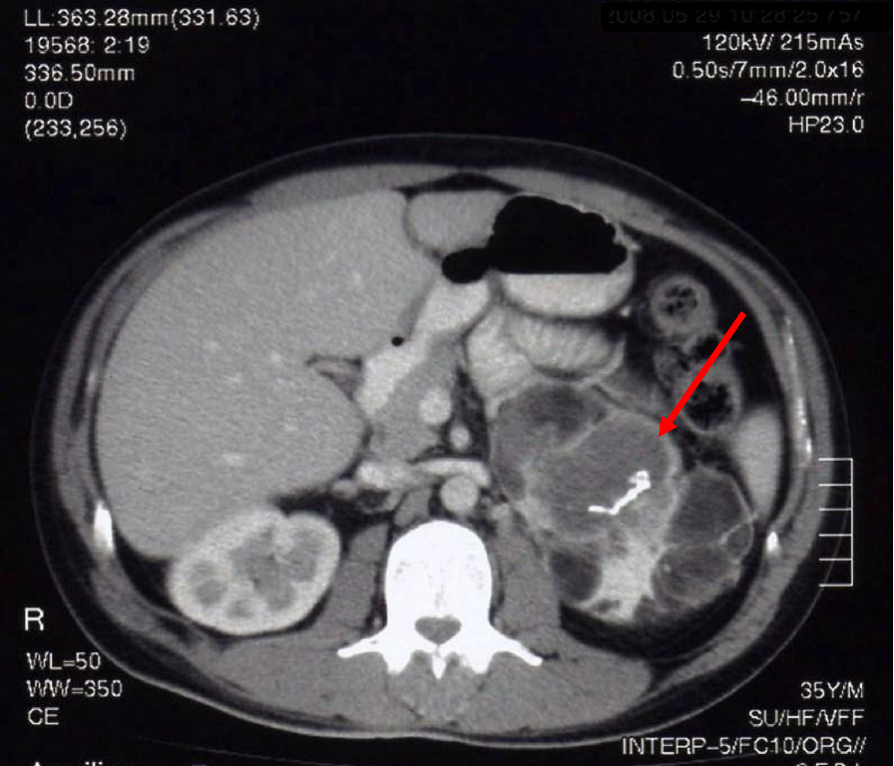
**Abdominal computed tomography scan.** Abdominal computed tomography scan before surgery showing the very enlarged and heterogeneous left adrenal gland (*arrow*) with soft tissue, fat and calcium attenuation.

An abdominal computed tomography scan identified a voluminous mass (diameter >12cm) with relatively well-defined limits but the composition was heterogeneous with a predominance of lipid attenuation and multiple areas of calcification, corresponding to the patient’s left adrenal gland. His right adrenal gland was also enlarged (diameter 6cm) and was also heterogeneous (Figure 1).

The scrotal sonogram showed slightly enlarged testicles with hyperechogenic and heterogenous nodules (diameter 4.5cm). Microscopic analysis of three surgical biopsy specimens (1cm each) revealed nodular hyperplasia of ectopic adrenal cells. The testicular tissue adjacent to the nodules showed reduced tubular diameter, hyalinization and peritubular fibrosis. Immunohistochemistry was positive for vimentin, inhibin, and actin and negative for cytokeratins AE1-AE3, placental alkaline phosphatase (PLAP), CD30, chromogranin and synaptophysin.

Based on the evidence of ineffective steroidogenesis and because malignancy could not be ruled out, bilateral adrenalectomy was chosen. No complications occurred during the surgery or the immediate post-operative period. Together, the excised masses weighed nearly 0.5kg (left, 382g; and right, 44g). The pathological examination revealed cortical nodular hyperplasia with bilateral myelolipomas. We repeated the cosyntropin stimulation test six months after surgery (Table 3), at 16 hours after the previous administration of oral hydrocortisone.

**Table 3:** Results of the cosyntropin stimulation test after bilateral adrenalectomy (basal, 30 and 60 minutes post-cosyntropin)

	**RV ♂**	**0 min**	**30 min**	**60 min**
**ACTH**	0–46pg/mL	4766	3130	2224
**Cortisol**	4.3–23μg/dL	1.4	1.3	1.3
**17-OHP**	0.6–3.4ng/mL	35.9	44.30	34.7
**11-deoxycortisol**	<7.2ng/mL	6.2	6.7	6.4
**Delta-4-androstenedione**	0.6–3.1ng/mL	21.9	31.1	25.2
**DHEAS**	80–560μg/dL	<15.0	<15.0	<15.0
**Total testosterone**	241–827ng/dL	1054.3	1399.1	1266.7

ACTH, Adrenocorticotropic hormone; DHEAS, Dehydroepiandrosterone-sulfate; min, Minute; 17-OHP, 17-hydroxyprogesterone; RV ♂, Reference values for the male population.

After informed consent was obtained from the patient, a genetic study was performed with deoxyribonucleic acid (DNA) extraction from whole blood followed by polymerase chain reaction and direct genetic sequencing techniques. Two distinct mutations affecting the *CYP21A2* gene were detected: heterozygous R124H (moderate defect); and homozygous R356W (severe defect). The last could be a true homozygous mutation or result from amplification of one of the alleles.

Before discharge from the Endocrine and Metabolic Department, the patient was prescribed hydrocortisone (20mg in the morning and 10mg in the afternoon) and fludrocortisone (100μg a day in the morning). Two months after surgery the cutaneous hyperpigmentation had almost disappeared and testicular nodules were significantly smaller. Since discharge he has not necessitated an emergency room visit.

## Discussion

The present patient was diagnosed with CAH in the neonatal period after a life-threatening episode, which was suggestive of a severe salt-wasting form of the disease. He was immediately started on daily replacement therapy with glucocorticoids and mineralocorticoids and compliance during childhood must have been acceptable, because normal pubertal development and adult stature were achieved. During adulthood, medical follow-up and self-administered therapy were irregular. He presented to the Emergency department with acute adrenal insufficiency, precipitated by an infectious disease.

Complete baseline endocrine evaluation (before beginning steroid replacement) revealed absolute cortisol deficiency, with elevated ACTH. The concentration of 17-OHP was 60 times the upper limit of normal, whereas 11-deoxycortisol was normal to high, which was unexpected. Renin was slightly elevated and aldosterone was normal. There was increased androgen production; androstenedione was very high. However, the total and free testosterone levels were normal and dehydroepiandrosterone-sulfate (DHEAS) was paradoxically low. This finding may be explained by the chronic stimulation of 3-β-hydroxysteroid dehydrogenase, resembling the physiologic response in chronic stress. We found only two other case reports in the literature which included the hormonal profile of male patients with CAH due to 21-OH deficiency and found low DHEAS, as was the case of our patient [7,8]. Here, the high to normal 11-deoxycortisol levels, the increased deoxycortisol to cortisol ratio, and the increased androstenedione with low levels of DHEAS indicate abnormal steroidogenesis and were the first clues of an extra-adrenal origin. The gonadotropins were suppressed, probably because of the inhibitory effect of the elevated adrenal sex steroids, suggesting that the circulating testosterone was adrenal in origin.

The cosyntropin stimulation test is the gold standard for determining the severity of disease [3]. It is performed by intravenous injection of a 125μg or 250μg bolus of cosyntropin, an ACTH-analogue, and measuring baseline and stimulated levels of 17-OHP [6]. Patients with the salt-wasting form have the highest post-stimulation 17-OHP levels (over 100,000ng/mL), those with the simple virilizing form have somewhat lower levels and patients with non-classical disease have lower levels (1500 to 10,000ng/mL) [3,5].

In our case the peak value of 17-OHP was 20,400ng/dL, consistent with CAH due to moderate 21-OH deficiency [3]. A 17-OHP which remains almost unchanged after stimulation has been described in other case reports when values at baseline were already very high [6,7,9]. It suggests that the adrenal cells were already maximally stimulated by chronically elevated ACTH and is corroborated by the nearly absent elevation of cortisol. In the present case there was an elevation of delta-4-androstenedione and DHEAS about one and a half times the baseline level. Levels of DHEAS remained below normal. There was a paradoxical increase of 11-deoxycortisol (the peak was almost triple that of the baseline value). Testosterone also increased in response to the ACTH analogue.

The results of the cosyntropin test performed after the adrenalectomy were indicative of a partial contribution of the adrenal cells in the adrenal cortex and testicles compared with the results found in the previous test. Cortisol levels remained nearly unchanged upon stimulation. The pattern of increased 11-deoxycortisol and decreased DHEAS was repeated but 17-OHP was markedly lower, probably indicating that the former two had primarily originated in the testicles and the latter in the adrenal glands. The fact that 17-OHP is still well above the upper limit of normal suggests that the ectopic testicular cells also have 21-OH deficiency.

The adrenal myelolipoma is a rare benign tumor consisting of mature adipose cells and hematopoietic tissue [6]. Prolonged stimulation of the adrenal cortex by ACTH seems to be implicated [6,8]. A review of the literature by Mermejo *et al*. in 2010 [8] found reports of 26 cases of myelolipoma associated with CAH. Of these, the majority was secondary to 21-OH deficiency and the patients were either untreated or had medication withdrawn for a long time. Several mechanisms have been proposed to explain the origin of myelolipomas. These include the presence of embryonic bone marrow rests in adrenal tissue or metaplasia of adrenocortical cells [7,9]. The majority of cases are benign. Surgical excision is advocated in the case of large or growing lesions (above five cm) that have heterogeneity, hemorrhage or other suspicious features, and in cases refractory to medical management [4,10].

The adrenal glands in the present case are remarkable for their dimension, weight, and heterogeneity, which raised concern about the possibility of malignancy and mass effect over the surrounding abdominal structures. In the review by Mermejo *et al*. [8] there were only seven reports in the literature of equally enlarged or larger adrenal glands as a result of CAH due to 21-OH deficiency. Recently, McGeoch *et al*. [11] reported a case of giant bilateral adrenal myelolipomas in a man with CAH.

Another curious aspect is the marked asymmetry in size of the tumors. Other recent case reports have described a much larger mass on the left side [6,9]. We speculate that it is due to restrained growth due to the proximity of the liver.

The testicular masses of the present patient were large, hyperechogenic and markedly heterogeneous. The tumor markers for germinative tumors (CEA, α-FP, β-HCG) were negative. There was massive deposition of collagen inside the seminiferous tubules, which is a general finding in end-stage testicular damage. The diagnosis of nodular bilateral hyperplasia of ectopic adrenal cells was made in the same tissue samples. The immunological stain was positive for actin and vimentin, markers for mesenchymatous tissue, as well as for inhibin, a marker for cells of stroma or sexual cord tumors, which could indicate a Leydig cell tumor. The stain was negative for AE1 and AE3, epithelial tumor markers, PLAP, which excluded the hypothesis of seminoma, CD30, a marker of embrionary carcinoma and also chromogranin or synaptophysin.

Patients with CAH and poorly controlled disease sometimes present with testicular masses representing adrenal rest tissue [12]. In prenatal life, the adrenal glands develop in the vicinity of the gonads. Adrenal cortical tissue may adhere to the gonad and descend along the course of the supplying arteries. Similar testicular lesions can be found in patients with other conditions characterized by elevated levels of ACTH, such as Addison’s disease and Cushing’s syndrome [13]. The estimated prevalence of adrenal rest nodules in patients with CAH is high, ranging from 24% to 95% [12-15]. These lesions are multifocal, bilateral, hypoechoic and well defined [12]. Nodules gradually expand and destroy the surrounding parenchyma and can lead to obstruction of the seminiferous tubules, resulting in deficient spermatogenesis and testosterone synthesis [13]. In addition, spermatogenesis is suppressed because gonadotropin secretion is inhibited by the elevated circulating androgens [15].

The genetic analysis revealed two different mutations: heterozygous R124H, responsible for a moderate defect and “apparent” homozygous 356W, causing almost undetectable residual enzymatic activity. The geneticist discussed as more probable the possibility of “compound heterozigoty”, the condition of having two heterogeneous recessive alleles in one particular locus causing genetic disease in a heterozygous state. In this case one allele would express the moderate and the other the severe mutation. In this case the phenotype would be defined by the milder mutation, and this agrees with the clinical manifestations of our patient.

## Conclusions

The diagnosis of CAH was established early in life, after what could be interpreted as a salt-wasting crisis. However, achievement of a normal adult stature and clinical stability despite irregular medical compliance is suggestive of a milder form. Clinical, analytic and genetic data combined, the diagnosis of simple virilizing 21-OH deficiency seems more probable.

Irregular medical compliance, low daily doses of hydrocortisone and inadequate stress adjustment of glucocorticoid dosage during acute infection resulted in irreversible consequences, ultimately resulting in major abdominal surgery and adrenal insufficiency. The masses in the adrenals and testicles resulted from chronically elevated ACTH and stimulated growth of adrenocortical cells. The basal and post-cosyntropin steroid profile, before and after adrenalectomy, revealed an unexpected pattern, suggesting significant contribution of the testicular adrenal cells to the steroidogenesis.

## Consent

Written informed consent was obtained from the patient for publication of this case report and accompanying images. A copy of the written consent is available for review by the Editor-in-Chief of this journal.

## Abbreviations

ACTH: Adrenocorticotropic hormone; α-FP: alpha-fetoprotein; β-HCG: beta-human chorionic gonadotropin; CAH: Congenital adrenal hyperplasia; CEA: Carcinogenic embryonic antigen; DHEAS: Dehydroepiandrosterone-sulfate; 21-OH: 21-hydroxylase; 17-OHP: 17- hydroxyprogesterone; PLAP: placental alkaline phosphatase.

## Competing interests

The authors declare that they have no competing interests.

## Authors’ contributions

FF, JM and SV provided medical care to the patient during in-patient admission, performed the functional tests and analyzed their results and drafted the manuscript. JM is the main care provider of the patient and has maintained follow-up consultations in the out-patient department. IC is the head of department. She participated in the discussion of the results of the dynamic tests and reviewed the manuscript. RE was the main surgeon present during the bilateral adrenalectomy. GN performed bilateral surgical biopsy of the testicular nodules. All authors read and approved the final manuscript.

## Authors’ information

FF, MD, BEE, has been a resident of Endocrinology for the past five years, working in the Endocrine and Metabolic Department of Santa Maria Hospital, Lisbon, since 2008.

JM, PhD, BEE, is a specialist in endocrinology with many years of clinical experience and scientific investigation. He has a particular interest in the subject of adrenal pathology and secondary hypertension. He has been working in the Endocrine and Metabolic Department of Santa Maria Hospital, Lisbon, since 2005 and is a teacher at the Lisbon School of Medicine.

SV, MD, BEE, is a specialist in Endocrinology. She works in the Endocrine and Metabolic Department of Santa Maria Hospital, Lisbon since 2003. She is also a teacher at the Lisbon School of Medicine and is currently enrolling in a PhD degree program.

RE, MS, is a very skilled surgeon with extensive experience in abdominal surgery. He has been working for the past 15 years in the Surgery Department of Santa Maria Hospital, Lisbon.

IC, PhD, BEE, has been the head of the Endocrine and Metabolic Department of Santa Maria Hospital since 2006. She has developed extensive clinical and scientific work in the field of obesity.
